# *H. pylori* Outer Membrane Protein Antibody Response, MHC/HLA-DMA Methylation, and Gastric Cancer Risk: A Multi-Layer Evidence Integration Study Using Mendelian Randomization

**DOI:** 10.3390/genes17091125

**Published:** 2026-09-15

**Authors:** Yangxi Fu, Zheng Wang, Shan Liu, Lin Liu, Xudong Tang

**Affiliations:** 1 Graduate School, Beijing University of Chinese Medicine, Beijing 100029, China; 20240941436@bucm.edu.cn; 2 Institute of Digestive Diseases, Xiyuan Hospital of China Academy of Chinese Medical Sciences, Beijing 100091, China; wz906502886@hotmail.com (Z.W.); llliver@126.com (L.L.); 3 Beijing Traditional Chinese Medicine Research Institute Co., Ltd., Beijing 100011, China; liush0403@126.com

**Keywords:** *Helicobacter pylori*, outer membrane proteins, gastric cancer, HLA-DMA, DNA methylation, Mendelian randomization

## Abstract

**Background:** *Helicobacter pylori* (*H. pylori*) infection is a well-established risk factor for gastric cancer; however, evidence on the associations between antigen-specific antibody traits, host methylation, and gastric cancer risk remains limited. **Methods:** Using a forward two-sample Mendelian randomization (MR) design, we integrated methylation quantitative trait loci (mQTLs), external cohorts, The Cancer Genome Atlas–Stomach Adenocarcinoma (TCGA-STAD), and functional annotations through a three-stage framework (antibody trait screening, CpG prioritization, and external replication) to evaluate a two-step pathway from host antibody response to gastric cancer via DNA methylation. **Results:** Among six *H. pylori* antigens, antibody response to outer membrane protein (OMP) showed the strongest positive association with gastric cancer risk (OR = 1.186, 95% CI: 1.079–1.304, raw *p* = 4.08 × 10^−4^). CpG-level prioritization identified cg24290947 (annotated to HLA-DMA) as a methylation site linked to gastric cancer susceptibility, with an inverse MR association (beta = −0.2347, 95% CI: −0.3980 to −0.0713, raw *p* = 0.00487). External exploration showed that cg24290947 methylation was elevated in *H. pylori*-positive gastric mucosa; however, its direct tumor–normal difference in a 12-pair cohort was not significant, although regional methylation perturbations were observed. HLA-DMA and MHC-II expression were consistently higher in tumor versus normal tissues across three cohorts, whereas TCGA-STAD did not support a stable linear relationship between cg24290947 methylation and HLA-DMA/MHC-II expression; three of six composition-adjusted models showed nominal significance but were model-dependent. These signals were enriched in antigen processing and presentation pathways. **Conclusions:** cg24290947/HLA-DMA represents a hypothesis-generating regional methylation candidate for further investigation rather than a confirmed linear transcriptional regulator. The MR evidence is based on European-ancestry summary data; its generalizability and any clinical predictive value warrant independent evaluation.

## 1. Introduction

Gastric cancer is a malignancy with one of the highest incidence and mortality rates worldwide. According to GLOBOCAN 2022 data, there are over 968,000 new cases and approximately 660,000 deaths from gastric cancer annually worldwide [1]. The disease has an insidious onset, often presenting no obvious symptoms in the early stages. Most patients are diagnosed at an advanced stage, making treatment challenging and significantly reducing the five-year survival rate. Public awareness and participation in gastric cancer screening remain generally low [2]. Furthermore, traditional serum markers such as CEA, CA19-9, and CA72-4 exhibit limited sensitivity and specificity during the early stages [3], posing a major technical bottleneck for early gastric cancer detection.

*H. pylori* is the most important controllable risk factor for gastric cancer and is classified as a Group I carcinogen by the International Agency for Research on Cancer. Although the global prevalence of *H. pylori* infection has declined in adults (from 52.6% before 1990 to 43.9% during 2015–2022), it remains as high as 35.1% in children and adolescents [4]. In China, *H. pylori* infection is associated with risk ratios of 5.94 and 3.06 for non-cardia and cardia gastric cancer, respectively [5]. *H. pylori* antigens, including Cytotoxin-associated antigen A (CagA), Vacuolating cytotoxin (VacA), Chaperonin GroEL (GroEL) and OMP, exhibit differential carcinogenic potentials and population-specific effects [6,7]. Studies have shown that in East Asian populations, OMP exhibits the strongest effect on non-cardia gastric cancer risk, with a 2.66-fold increased risk relative to controls, surpassing the traditional virulence factor CagA at 2.20-fold. In European populations, however, only CagA and GroEL are significantly associated, conferring 5.83-fold and 3.66-fold increased risks, respectively, whereas OMP shows no significant effect [7], highlighting the complexity of host–pathogen interactions.

Aberrant DNA methylation, particularly promoter CpG island hypermethylation, plays a critical role in *H. pylori*-induced malignant transformation of gastric mucosal cells [8]. Promoter CpG island hypermethylation frequently leads to transcriptional silencing of tumor suppressor genes [9]. *H. pylori* infection is one of the most important environmental factors inducing aberrant DNA methylation in gastric mucosa. Maekita et al. reported that methylation levels of multiple CpG islands were significantly elevated (5.4- to 303-fold) in *H. pylori*-positive individuals [10]. Niwa et al. further demonstrated in a Mongolian gerbil model that *H. pylori*-induced inflammation drives de novo CpG island methylation in gastric epithelial cells [11]. Moreover, *H. pylori*-induced aberrant DNA methylation can establish an “epigenetic field defect” in gastric mucosa, with methylation levels at certain loci remaining elevated even after *H. pylori* eradication [12,13].

**Figure 1:**
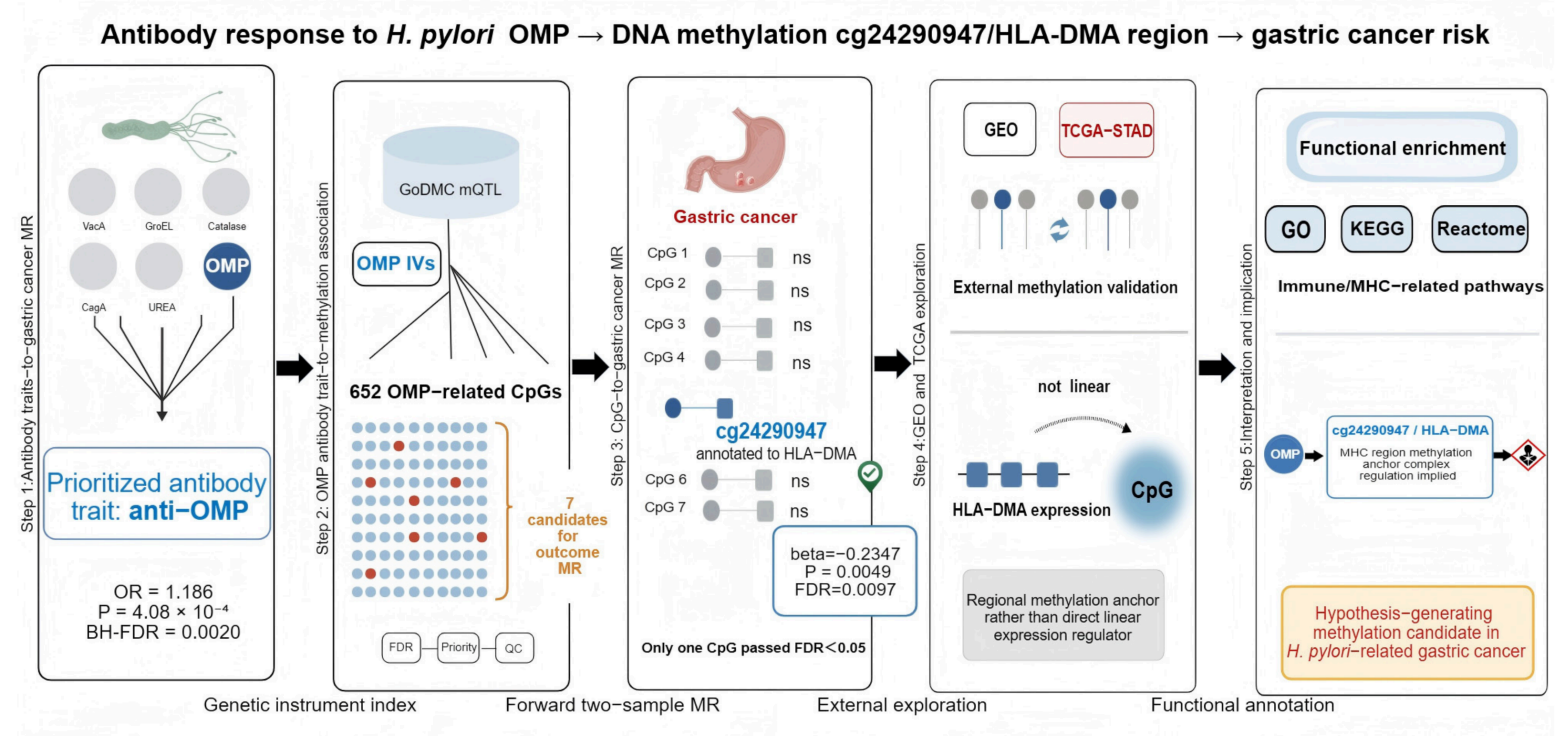
Study design and analytical workflow. The workflow integrates antibody-level MR, exploratory lead antibody trait-associated CpG prioritization, multi-stage CpG-to-gastric-cancer evaluation, contextual assessment in external methylation and expression cohorts, a within-sample TCGA-STAD methylation-expression subanalysis, and targeted functional annotation.

Although observational studies have revealed close associations among *H. pylori* infection, DNA methylation, and gastric cancer, the existing evidence is largely limited to descriptive correlational analyses and lacks a genetically informed framework [10,13,14]. Specifically, previous studies have two major limitations. First, most have treated *H. pylori* infection as a homogeneous exposure, failing to distinguish the effects of specific virulence antigens (e.g., CagA, OMP) on host epigenetic modifications; consequently, whether aberrant methylation at specific CpG sites is genetically associated with particular antigen subtypes remains unclear. Second, a complete mediation chain from antigen-specific immune response to gastric cancer via DNA methylation has not been systematically established, leaving the mediating role of epigenetic alterations in *H. pylori*-associated gastric carcinogenesis speculative. To address these limitations and to distinguish observational antigen–antibody associations from genetically proxied antibody responses, this study employs a forward two-sample MR approach, using genetic variants associated with host antibody responses to *H. pylori* antigens as exposure instruments and mQTLs as mediator instruments. This framework evaluates a two-step pathway from antibody traits to DNA methylation to gastric cancer and systematically assesses the associations between antibody-related methylation signals and gastric cancer risk. The specific aims include: (1) identifying the *H. pylori* antigen whose antibody response is most significantly associated with gastric cancer risk; (2) identifying CpG sites associated with this antibody response and evaluating their genetic associations with gastric cancer through multi-stage outcome assessment; and (3) exploring tissue-level support for methylation signals in the candidate regions using external independent data. It is important to note that the present MR analysis evaluates genetically proxied antibody responses, which provides a distinct line of evidence from earlier observational reports of antigen–antibody associations at the population level. The study framework is shown in Figure 1.

## 2. Materials and Methods

### 2.1. Study Design and Analytical Framework

The study combined publicly available summary-level genetic data, mQTLs, and external multi-layer data to construct a hierarchical evidence framework, following the logic of forward two-sample MR and a two-step MR design. The analytical workflow comprised four stages. First, MR analyses were performed for six *H. pylori* antibody traits against gastric cancer risk to screen for the lead antibody trait for subsequent methylation analyses. Second, using the lead antibody trait as an anchor, we identified its associated CpGs by integrating GoDMC mQTL data, and formed the exploratory discovery-phase candidate set based on lead trait–CpG statistical support, retention status in the candidate selection process, and genomic annotation. Third, we performed CpG-to-gastric cancer MR on the candidate CpGs to evaluate their outcome-level genetic associations with gastric cancer risk. Fourth, using external Gene Expression Omnibus (GEO) methylation and expression datasets, TCGA-STAD integrative analysis, and directed functional annotation, we evaluated the methylation characteristics of the candidate region, the expression context of MHC-II antigen presentation, and related pathway enrichment in the gastric cancer tissue setting. The upstream antibody trait layer served to determine the *H. pylori*-related exposure entry point; the CpG layer served to identify host methylation signals associated with the lead antibody trait; the outcome-layer MR served to quantify the genetic associations between candidate CpGs and gastric cancer risk; and external exploratory analyses provided independent tissue-level evidence. Outcome-layer MR and external evidence were not used in the discovery-phase CpG screening, but were independently applied in the subsequent integrated evidence classification.

This study is reported in accordance with STROBE-MR guidelines; the completed checklist is provided in the Supplementary STROBE-MR Checklist.

### 2.2. Data Sources for H. pylori Antibody Traits GWAS, mQTL and Gastric Cancer

In this study, the *H. pylori* antibody traits used as exposures in MR analyses denote genetically proxied antibody responses, rather than direct measurements of antigen exposure, bacterial colonization, or protein dose. Summary-level GWAS data for antibody traits targeting *H. pylori* antigens were obtained from the IEU OpenGWAS/MR-Base platform, all derived from European populations. The six included *H. pylori* antigen–antibody traits and their corresponding OpenGWAS IDs were as follows: CagA (ebi-a-GCST90006911, *n* = 985), Catalase (ebi-a-GCST90006912, *n* = 1558), GroEL (ebi-a-GCST90006913, *n* = 2716), OMP (ebi-a-GCST90006914, *n* = 2640), UREA (ebi-a-GCST90006915, *n* = 2251), and VacA (ebi-a-GCST90006916, *n* = 1571). In the originating antibody GWAS, quantitative antibody Mean Fluorescence Intensity (MFI) analyses were restricted to participants exceeding the relevant seropositivity threshold; the quantitative phenotypes were standardized to mean of 0 and standard deviation of 1 and analyzed using a fastGWA mixed model including sex, age, assessment centre and the first 20 principal components [15]. Gastric cancer outcome data were obtained from ebi-a-GCST90018849, comprising 1029 cases and 475,087 controls (total *n* = 476,116). Data extraction, harmonization, and analysis for the two-sample MR were performed following the MR-Base/TwoSampleMR framework [16].

Lead antibody trait-associated CpG discovery was conducted using mQTL summary data from the GoDMC public mQTL release [17]. GoDMC is a large-scale resource based on peripheral-blood DNA methylation, integrating data from 32,851 participants and covering 420,509 methylation sites. Lead antigen–antibody instruments were used as exposure instruments to evaluate their associations with CpG methylation in the GoDMC data. GoDMC provides blood-derived genetic associations with methylation rather than gastric mucosa-specific mQTL estimates. Thus, the overall participant count of the resource does not represent the actual sample size analyzed for each genetic variant. Gastric tissue datasets were used as separate contextual evidence and were not interpreted as proof that the index blood mQTL is applicable to gastric mucosa. The sources, sample scope, and analytical purposes of the *H. pylori* antibody response GWAS, gastric cancer GWAS, mQTL data, and each external multi-layer dataset are detailed in Supplementary Table S1.

### 2.3. Instrumental Variable Selection and MR Analysis

This study involved three analytical layers. The first layer comprised MR analyses between *H. pylori* antibody traits and gastric cancer, used to compare the MR-based associations of the six *H. pylori* antibody traits with gastric cancer risk and to determine the lead antibody trait. The second layer involved lead antibody trait-associated CpG mQTL analyses, used to identify host DNA methylation signals associated with the lead antibody trait from the GoDMC-derived mQTL results. The third layer comprised CpG-to-gastric cancer MR analyses, used to evaluate the associations of retained candidate CpGs with gastric cancer risk. For this layer, exposure instruments were taken from CpG-associated records marked as clumped in the GoDMC public release. The first and third layers of MR analyses followed the same unified instrumental variable selection and quality control criteria; the second layer did not involve an independent instrumental variable selection process.

Antibody-level MR analysis: Instrumental variables were extracted from the exposure GWAS at a threshold of *p* < 5 × 10^−6^ and LD-clumped using an r^2^ threshold of 0.001 within a 10,000-kb window with a European reference panel. Exposure and outcome data were harmonized using action = 2 to align effect alleles, with allele-frequency information used when handling palindromic variants. The inverse variance weighted (IVW) method [16] was used as the primary estimator. MR-Egger, weighted median, simple mode, and weighted mode estimates were used as sensitivity analyses when the available instrument number permitted. Heterogeneity was assessed using Cochran’s Q test, directional horizontal pleiotropy using the MR-Egger intercept test, and MR-PRESSO global testing when applicable. Instrument strength for traceable instrument sets was assessed using F = (beta_exposure/SE_exposure)^2^. Supplementary Table S2 reports the counts of variants meeting *p* < 5 × 10^−6^ in the retained source GWAS VCFs, historical post-clumping counts, and final MR counts.

CpG-to-gastric cancer MR analysis: Exposure instruments were taken from CpG-associated records marked as clumped in the GoDMC public release; no new *p* < 5 × 10^−6^ selection or re-clumping with the antibody-level parameters was performed for this layer. CpG exposure records were harmonized with the gastric cancer GWAS using action = 2. For multi-instrument analyses, IVW was used as the primary estimator; for single-instrument analyses, the Wald ratio was applied. MR-Egger, weighted median, simple mode, and weighted mode estimates, as well as Cochran’s Q, MR-Egger intercept, and MR-PRESSO results, were obtained when the available instrument number and source data permitted.

### 2.4. Lead Antibody Trait-Associated CpG Prioritization

This section describes only the discovery-phase CpG prioritization; subsequent CpG-to-gastric cancer MR or external evidence results were not used as screening criteria.

Detected CpGs were defined as unique CpG sites detectable in the lead antibody trait–CpG association table. FDR-supported CpGs were defined as those with Benjamini–Hochberg false discovery rate (FDR) < 0.05 in the lead antibody trait–CpG association analysis. Retained candidate CpGs were defined as unique CpG sites meeting exploratory tier-based prioritization criteria, which allowed both FDR-supported and nominally significant associations to be retained according to tier-specific requirements for instrument number, annotation, and available quality indicators. For each CpG, the lead association estimates, standard errors, nominal *p*-values, and FDR were first compiled and then retained by unique CpG ID to form the retained candidate CpG set. The complete screening rules, handling of missing diagnostics, instrument inclusion criteria, direction consistency thresholds, sensitivity diagnostic cutoffs, and the full list of all 652 evaluated OMP antibody trait–CpG associations are provided in Supplementary Methods and Supplementary Table S3A,B. These discovery-stage filters were applied independently of the subsequent outcome-layer MR results. Subsequent evaluation proceeded along two non-mutually exclusive branches: (i) a strict HLA/MHC source-annotated branch, used to flag CpGs localized to the HLA/MHC region; and (ii) a CpG outcome-layer evaluation set, used for two-sample MR of CpGs to gastric cancer. Outcome-layer MR and external evidence were independently evaluated in subsequent phases and used only for integrated evidence classification, not for the discovery-phase screening of retained candidates.

### 2.5. External Methylation Exploration

For external methylation exploration, three gastric mucosa- or gastric cancer-related GEO datasets were included [18]. GSE99553 was used for the *H. pylori* exposure-related methylation exploration layer, comparing *H. pylori*-positive versus *H. pylori*-negative gastric mucosa samples. GSE164988 was used for the direct CpG-level tumor-normal methylation exploration layer, comparing gastric cancer versus paired adjacent normal tissues. GSE30601 was used for the gene annotation-based regional methylation exploration layer, comparing gastric tumor versus non-malignant gastric tissue samples. GSE99553 and GSE164988 were based on the Illumina Human Methylation 450 platform, while GSE30601 was based on the Illumina Human Methylation 27 platform.

All GEO methylation analyses were performed using the processed methylation beta-value matrices provided in the GEO series matrix files. In the present study, no unified re-normalization was performed on the raw IDAT files from different datasets, nor were beta values merged across platforms. Prior to analysis, coverage of the candidate CpGs in the corresponding platforms was examined, and detectable CpGs were subjected to probe-level evaluation. For datasets in which the index CpG was not covered by the platform (e.g., GSE30601), regional evaluation was restricted to CpGs that were detectable in the platform annotation file and annotated to HLA-DMA, HLA-DMB, or BRD2. This restriction was based on the platform gene annotation and did not employ a fixed physical distance window.

For GSE164988, paired samples were analyzed using the two-sided paired Wilcoxon signed-rank test. For GSE99553 and GSE30601, unpaired comparisons were performed using the two-sided Wilcoxon rank-sum test. Methylation effects were expressed as delta beta values, with the direction defined as the tumor group or *H. pylori*-positive group minus the corresponding reference group.

### 2.6. External Expression Exploration and MHC-II Signature

For external expression exploration, three GEO gastric cancer expression cohorts were included [18], and the processed expression matrices provided by each dataset were used. GSE29272, based on the GPL96 Affymetrix Human Genome U133A Array, contained 134 pairs of tumor-normal expression samples. GSE54129, based on the GPL570 Affymetrix Human Genome U133 Plus 2.0 Array, contained 111 gastric cancer tissues and 21 non-cancerous gastric tissues. GSE66229, based on the GPL570 Affymetrix Human Genome U133 Plus 2.0 Array, contained 300 tumor and 100 normal samples. Each dataset was analyzed independently within its own platform; raw expression values were not merged across platforms.

The MHC-II antigen-presentation gene panel was based on HLA-DM peptide loading, MHC class II antigen presentation, invariant chain, and MHC-II transcriptional regulation, and was used to evaluate pathway-level expression context in gastric cancer tissues. The included genes were HLA-DMA, HLA-DMB, HLA-DRA, HLA-DRB1, HLA-DPA1, HLA-DPB1, CD74, and CIITA. HLA-DMA is both the annotated gene for cg24290947 and a member of the HLA-DM complex; HLA-DMB is the HLA-DM complex partner; HLA-DRA/HLA-DRB1/HLA-DPA1/HLA-DPB1 represent classical MHC-II molecules; and CD74 and CIITA represent the invariant chain and the MHC-II master regulator, respectively.

Probe-to-gene mapping was performed based on the corresponding GEO platform annotation. If multiple probes corresponded to the same gene, the median absolute deviation (MAD) was calculated across all samples in that dataset, and the probe with the highest MAD was selected to represent that gene. The MHC-II mean z-score signature was calculated independently within each dataset. Briefly, for detectable panel genes, z-score normalization was performed across all samples in that dataset, followed by the calculation of the mean of the available normalized gene expression values for each sample. Panel genes not detected by the platform were excluded from the signature calculation for that dataset; samples were not excluded because of the undetectability of a single panel gene. Paired data were analyzed using the two-sided paired Wilcoxon signed-rank test, and unpaired data were analyzed using the two-sided Wilcoxon rank-sum test.

### 2.7. TCGA-STAD Combined Analysis

The TCGA-STAD methylation-expression combined analysis included 354 primary tumor samples with matched methylation and expression data [19]; i.e., the same TCGA sample identifier had both HM450 methylation beta-values and RNA expression profiles (representing within-sample methylation-expression matching, rather than a tumor-normal paired design). In this analysis, expression of the annotated gene for cg24290947 referred to HLA-DMA expression; expression of the MHC-II gene panel and the MHC-II signature score were also evaluated. In the unadjusted analyses, Spearman correlation was used as the primary method for continuous variables, with Pearson correlation also recorded for reference. Additionally, high-versus low-methylation group comparisons were performed based on the median cg24290947 methylation beta-value.

For the composition-adjusted analyses, the TCGA-STAD full expression matrix (UCSC Xena, log2(count + 1)-transformed, mapped to gene symbols via gencode.v36 annotation, with duplicate genes aggregated by median) and ESTIMATE v1.0.13 were used to calculate ImmuneScore, StromalScore, and ESTIMATEScore. Tumor purity derived from ESTIMATE was calculated according to the formula reported in the literature [20], and was used solely as a sensitivity indicator for RNA-seq composition. After matching the expression matrix and methylation data by sample barcode, the intersection with the methylation-expression paired samples (*n* = 354) was used for the subsequent composition-adjusted models. Six intercept-included OLS models were fitted separately for HLA-DMA expression or MHC-II signature ~ cg24290947 methylation + ImmuneScore, StromalScore, or TumorPurity; highly correlated composition scores were not included simultaneously in the same model. Each model was analyzed based on its respective complete set of samples, and the regression coefficient for the methylation term, model standard error, two-sided *p*-value, and adjusted R^2^ were reported.

### 2.8. Targeted Functional Annotation

The study performed hypothesis-driven directed enrichment analysis. The input gene set consisted of two components: the predefined MHC-II antigen-presentation panel genes, and the regional genes annotated to candidate CpGs in the MHC/HLA region, specifically including HLA-DMA, HLA-DMB, HLA-DRA, HLA-DRB1, HLA-DPA1, HLA-DPB1, HLA-DQB1, HLA-DQB2, CD74, CIITA, BRD2, and GPSM3. It should be noted that the MHC-II expression signature panel described in Section 2.6, which was used for pathway scoring in the external expression cohorts, was narrower in scope. For the functional annotation input in this section, the regionally annotated genes of candidate CpGs in the MHC/HLA region were added to that panel; therefore, the input gene set for functional annotation was broader, and its composition was not entirely consistent with that of the signature panel, as the two served different purposes. The complete input genes, source categories, and inclusion criteria are provided in Supplementary Table S4.

Enrichment analysis was performed via the Enrichr API [21], using the following gene set libraries: GO_Biological_Process_2023 [22], KEGG_2021_Human [23], and Reactome_2022 [24]. The analyses used the default reference gene sets provided by each library, and no custom background gene set was submitted. The adjusted *p*-values returned by Enrichr were used as the FDR indicator, with FDR < 0.05 considered statistically supported. This analysis was a targeted enrichment for specific pathways, rather than a genome-wide unbiased exploration. From the Enrichr output, terms with FDR < 0.05 that were directly related to antigen processing/presentation, MHC-II peptide loading, adaptive immune response, or mucosal immune context were selected for presentation.

### 2.9. Statistical Analysis

MR analyses were performed using R 4.5.0, TwoSampleMR 0.7.0, and the MR-Base workflow, with specific parameters as documented for the corresponding antibody-MR calls. In the TwoSampleMR clump_data function, parameters were set as clump_kb = 10,000, clump_r^2^ = 0.001, and pop = “EUR”. The harmonise_data function was used with the default action = 2, which employs allele frequency information to assist in handling palindromic SNPs. For traceable instrument sets, F statistics were calculated as (beta_exposure/SE_exposure)^2^, with instrument sets clearly defined. R^2^ values were not calculated as exact variance explained could not be reliably reconstructed from the summary statistics. Table preparation, external cohort statistical analyses, and the TCGA-STAD methylation combined analysis were performed using Python 3.12.10 with the pandas, numpy, scipy, statsmodels, and matplotlib packages.

The analysis-specific tests are described in Section 2.5, Section 2.6 and Section 2.7; this section only summarizes the general statistical principles. All statistical tests were two-sided. For two-group comparisons, paired or unpaired non-parametric tests were selected according to the study design. Multiple comparisons were corrected using the Benjamini–Hochberg FDR method, with FDR < 0.05 considered the threshold for statistical significance, unless otherwise specified. Adjustment was applied within the corresponding analysis families rather than across different analysis families, and main-text MR findings are reported with effect estimates, 95% confidence intervals, and raw *p* values. TCGA-STAD composition-adjusted models were exploratory sensitivity analyses, and their *p*-values were interpreted only as nominal sensitivity evidence, not as independent criteria for positive confirmation.

## 3. Results

### 3.1. OMP Antibody Was Prioritized as the Leading H. pylori Antibody Trait Associated with Gastric Cancer Risk

**Figure 2:**
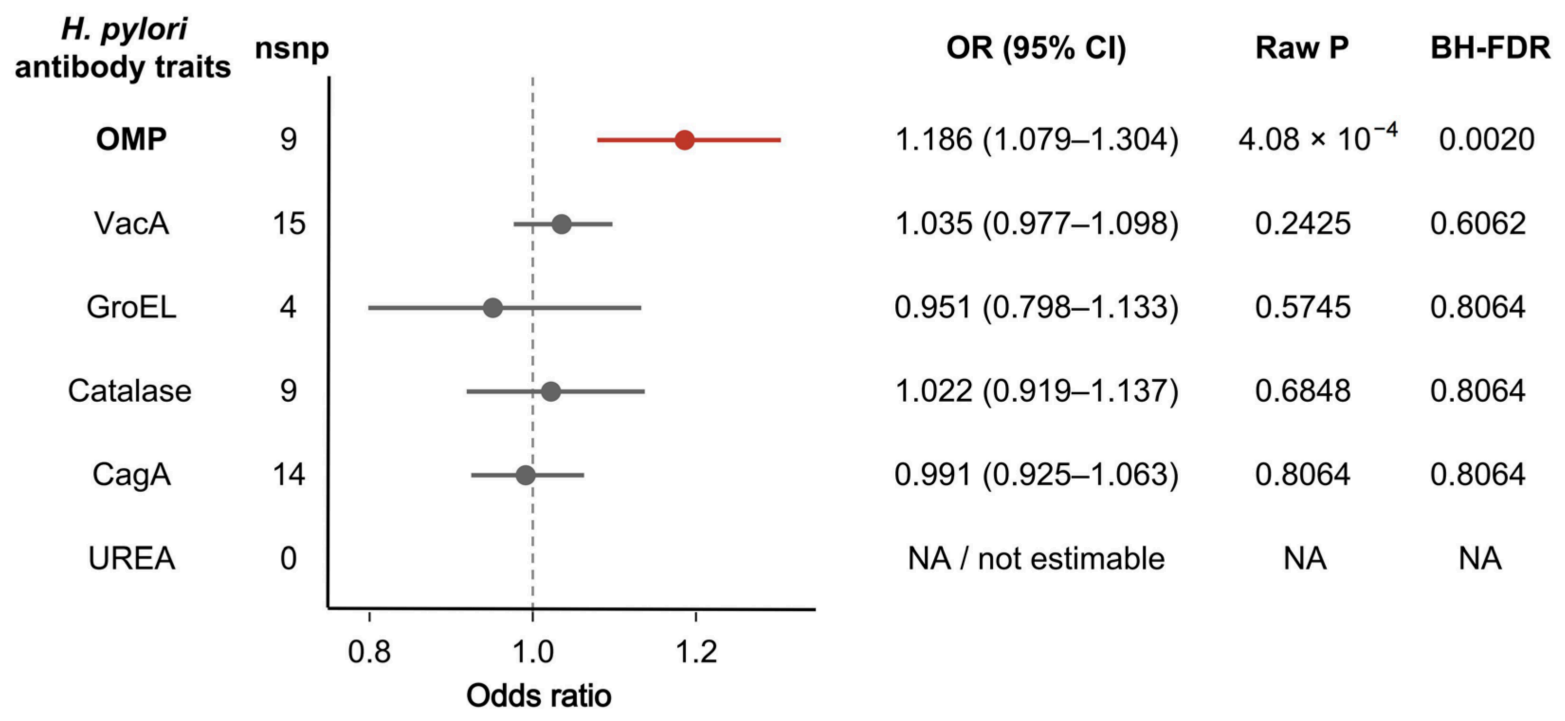
Forest plot of MR results for *H. pylori* antibody traits and gastric cancer risk. Points and horizontal intervals show odds ratios and 95% confidence intervals; displayed *p* values are raw (unadjusted) *p* values. BH-FDR-adjusted *p* values are shown alongside the estimates. The vertical reference line denotes OR = 1. OMP is highlighted in red as the lead trait; gray points represent the other four traits with estimable IVW results. UREA was not estimable.

Among the six *H. pylori* antibody traits, OMP showed the most robust positive association with gastric cancer risk (IVW OR = 1.186, 95% CI: 1.079–1.304, raw *p* = 4.08 × 10^−4^). After BH-FDR correction within the five-trait family among antibody traits with estimable IVW results, OMP remained significant (adjusted *p* = 0.0020) and was therefore prioritized as the lead antibody trait for subsequent CpG discovery analyses. No significant associations with gastric cancer risk were observed for the remaining antibody traits, including VacA, GroEL, Catalase, and CagA (all raw *p* > 0.05; Figure 2). UREA did not yield a valid IVW estimate owing to the absence of available SNPs after harmonization and was therefore not included in the primary analysis or FDR correction. Historical post-clumping/final MR counts for the six traits were 15/14 for CagA, 9/9 for Catalase, 5/4 for GroEL, 10/9 for OMP, 10/0 for UREA, and 15/15 for VacA. For the traceable OMP final instrument set, the minimum, median, and mean F statistics were 20.9222, 22.5142, and 24.7473, respectively. Sensitivity analyses supported the robustness of the OMP–gastric cancer association, with no significant heterogeneity detected (Cochran’s Q *p* = 0.2159) or directional horizontal pleiotropy (MR-Egger intercept *p* = 0.2247; MR-PRESSO global test *p* = 0.208); although these sensitivity analyses cannot completely rule out all bias, they provide no evidence of major violations. Full MR main results, multiple-method estimates, and sensitivity analyses are provided in Supplementary Table S5, and instrument-count and strength details are available in Supplementary Table S2.

### 3.2. OMP-Associated CpG Discovery Identified MHC/HLA-Region Candidates and Seven CpGs for Outcome-Layer MR

**Figure 3:**
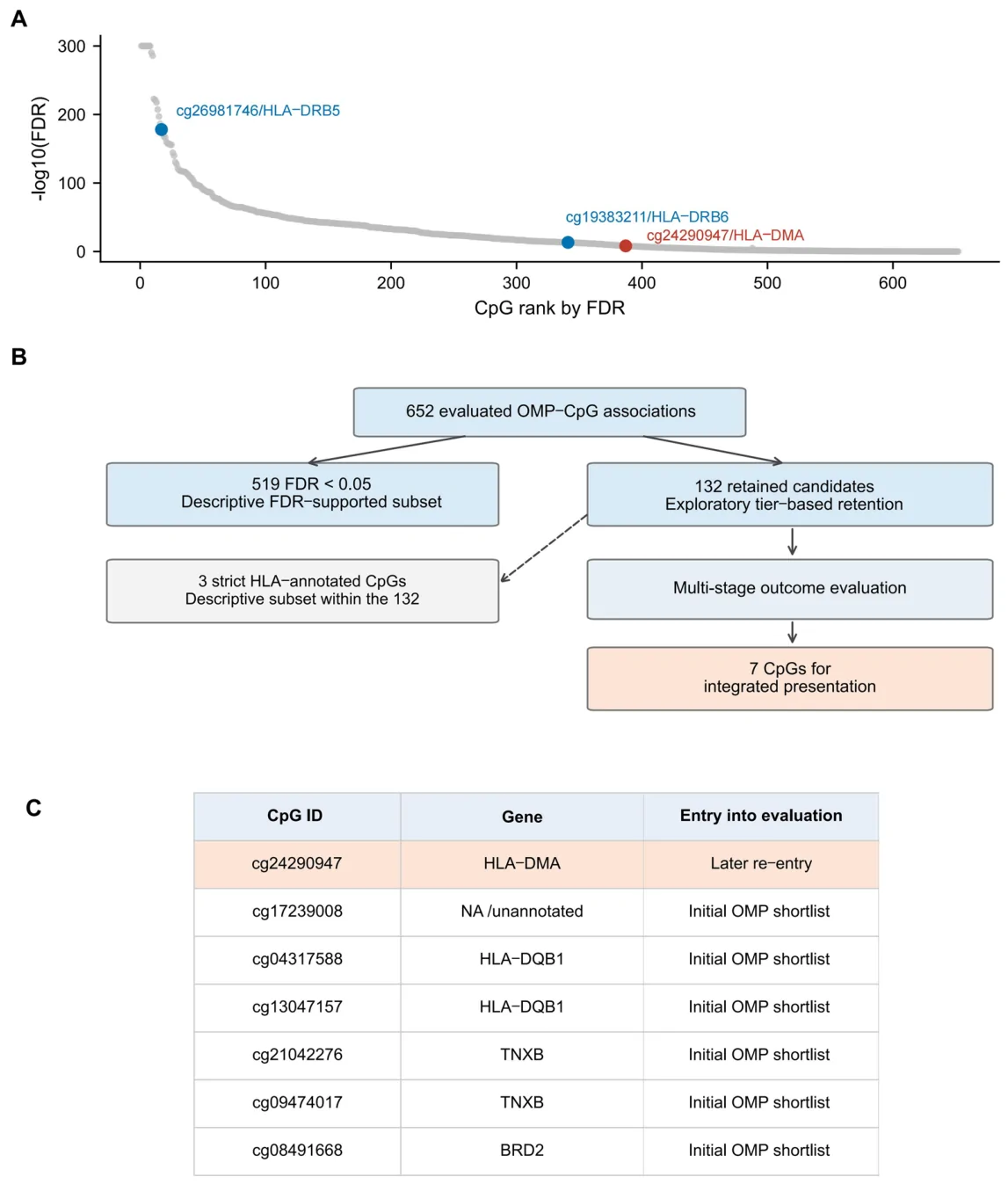
Discovery and candidate evaluation of OMP-associated CpGs. (**A**) Global distribution of 652 evaluated OMP-CpG associations ranked by BH-adjusted *p* values. Among the three highlighted CpGs, cg26981746/HLA-DRB5 and cg19383211/HLA-DRB6 are shown in blue, and cg24290947/HLA-DMA is highlighted in red. (**B**) Prioritization workflow: 652 evaluated associations→519 FDR-supported associations→132 retained candidates, with subsets for 3 HLA-annotated CpGs and 7 CpGs for integrated presentation. (**C**) Table of the seven CpGs with gene annotations and entry stages. NA/unannotated indicates the absence of a stable gene symbol in the relevant source annotation.

To identify host DNA methylation signals regulated by OMP-related genetic instruments and to screen CpGs for subsequent outcome-layer MR evaluation, we first performed OMP–CpG association analyses at the global CpG level. A total of 652 associated CpGs were detected, of which 519 reached FDR < 0.05 after multiple corrections. In the global distribution plot, MHC/HLA region sites were prominently highlighted, suggesting that this region harbors candidate methylation signals worthy of further evaluation (Figure 3A); three source-annotated HLA CpGs were also marked with colored dots as regional context examples. The above counts of 652 and 519 reflect specific features of the evaluated dataset rather than representing a sequential filtering funnel from 652 to 519 sites.

During the prioritization screening stage, we retained 132 candidates (109 Tier2 and 23 TierS) based on exploratory tier-based rules. This retained number is independent of the descriptive count of 519 FDR-supported associations, and retention itself does not imply that every candidate passed FDR or all sensitivity analyses. These 132 candidates entered two non-mutually exclusive evaluation branches: (i) a strict HLA/MHC source-annotated CpG branch (3 CpGs), used to flag CpGs located in the HLA/MHC region; and (ii) an evaluation set for outcome-layer MR, used for CpG-to-gastric cancer MR analyses. The outcome evaluation initially included 8 OMP-linked CpGs and 4 VacA comparators, with 3 additional OMP candidates added later, ultimately assembling 7 CpGs for integrated presentation. The overlap between the three HLA/MHC-annotated CpGs and the final seven CpGs was limited to cg24290947/HLA-DMA (Figure 3B), indicating that they arise from different filtering criteria and represent complementary rather than inclusion-based branches. Detailed information for the seven retained candidate CpGs (CpG ID, gene annotation, annotation status, and entry into evaluation) is presented in Figure 3C and Supplementary Table S6, where cg17239008 lacks a stable gene symbol in the core OMP–CpG association result table and is therefore denoted as “NA/unannotated.”

In summary, OMP-associated CpG signals were relatively abundant at the global level. Following FDR correction and tier-based prioritization, a candidate set for outcome-layer MR evaluation was obtained, while the strict HLA/MHC-annotated CpGs served as a regional localization branch to assist in subsequent integrated evidence classification.

### 3.3. CpG-to-Gastric Cancer MR Prioritized cg24290947/HLA-DMA as the Outcome-Layer Supported Candidate

**Figure 4:**
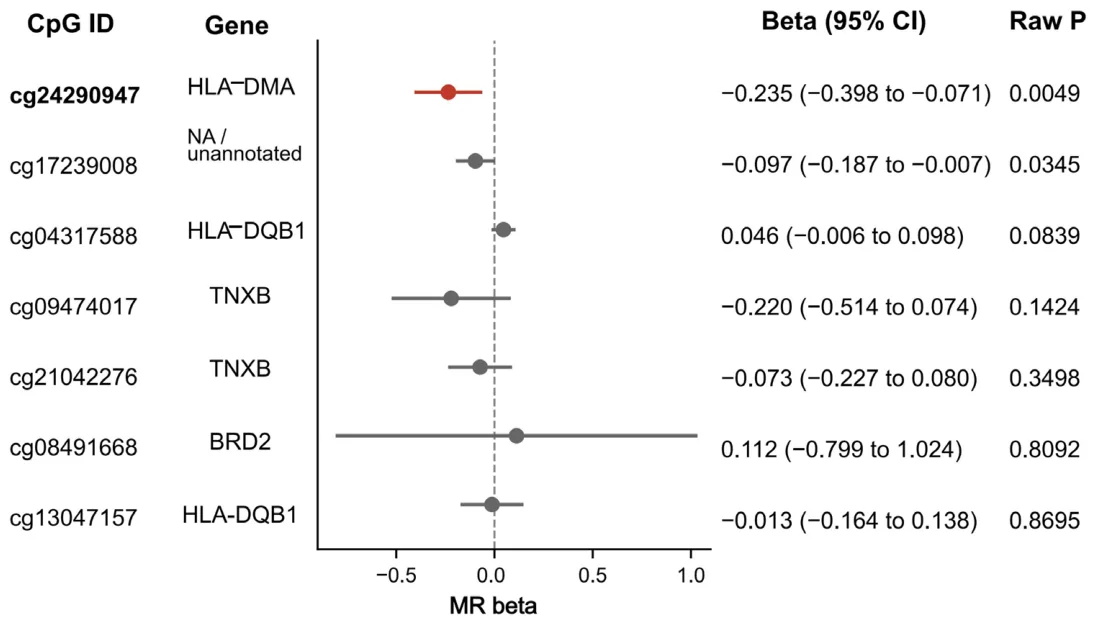
Forest plot of outcome-layer MR results for the seven candidate CpGs and gastric cancer risk. Points and horizontal intervals show beta estimates and 95% confidence intervals; displayed *p* values are raw (unadjusted) *p* values. The vertical reference line denotes beta = 0. cg24290947 is highlighted in red as the focus of integrated presentation; gray points represent the other six CpGs.

After identifying the seven retained CpGs, multi-stage gastric cancer outcome evaluations were performed on the candidate CpGs to identify a prioritized candidate supported by outcome-layer evidence. Among these, cg24290947/HLA-DMA showed the strongest genetic association (beta = −0.2347, 95% CI: −0.3980 to −0.0713, raw *p* = 0.0049; Figure 4); sensitivity analyses revealed no significant heterogeneity or directional horizontal pleiotropy (Cochran’s Q *p* = 0.5054; MR-Egger intercept *p* = 0.7320), and the five traceable instruments had minimum, median, and mean F statistics of 66.2075, 132.1568, and 165.7898, respectively. The remaining six CpGs did not reach the significance level of FDR < 0.05, although cg17239008 displayed a nominal signal at the *p*-value level (beta = −0.097, *p* = 0.0345), which did not remain significant after the corresponding stage-specific multiple-testing adjustment. Because the displayed CpGs originated from different stages of outcome evaluation, they were not analyzed under a common seven-test FDR family. The estimates and instrument QC for the seven displayed CpGs, along with the stage-specific adjusted *p* values and testing families for all 15 CpGs evaluated for gastric cancer, are reported in Supplementary Table S7A,B. Collectively, cg24290947 was the only CpG among the seven candidates that reached FDR < 0.05 in the outcome-layer MR analysis, suggesting that its genetic association with gastric cancer risk was the most robust. Therefore, it was positioned as the outcome-layer-supported candidate and advanced to subsequent integrated evidence evaluation.

### 3.4. External Methylation Datasets Provided H. pylori Exposure, Direct-Site, and Regional Methylation Evidence

**Figure 5:**
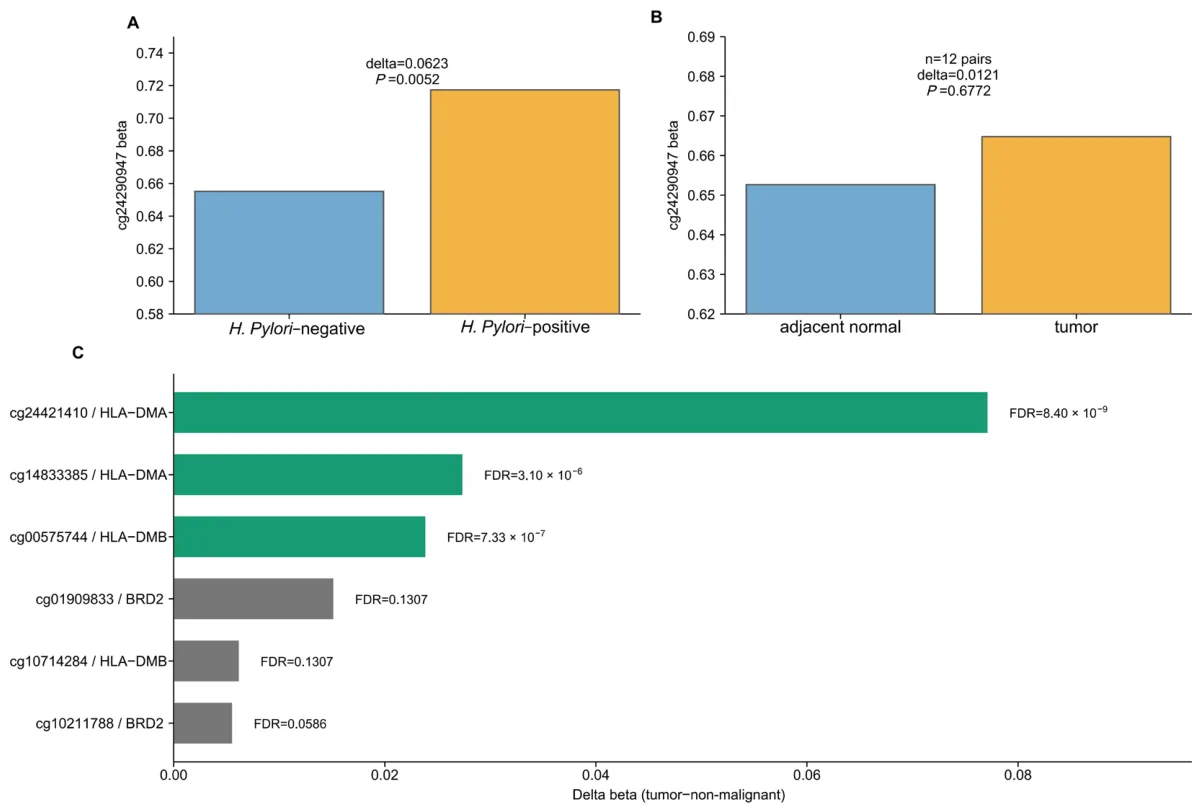
External methylation evidence for cg24290947 and HLA-DMA region. (**A**) Methylation difference at cg24290947 between *H. pylori*-positive and *H. pylori*-negative gastric mucosa in GSE99553. (**B**) Direct CpG-level assessment of cg24290947 in gastric cancer versus paired adjacent normal tissues in GSE164988. (**C**) Delta beta values and FDR for regionally covered CpGs annotated to HLA-DMA/HLA-DMB/BRD2 in GSE30601. GSE30601 did not cover cg24290947, so panel (**C**) reflects regional methylation context rather than direct CpG-level exploration of cg24290947.

To evaluate the tissue-level support for the candidate CpG cg24290947 and related regional signals in external independent datasets, exploration was performed at three levels: GSE99553 was used to explore *H. pylori* exposure-related methylation differences, GSE164988 for direct CpG-level tumor-normal differences, and GSE30601 for regional methylation signals (Figure 5).

GSE99553 revealed a methylation difference at cg24290947 between *H. pylori*-positive and *H. pylori*-negative gastric mucosa (delta beta = 0.0623, *p* = 0.0052). GSE164988, which examined direct CpG-level tumor-normal differences at cg24290947 in 12 paired gastric cancer/adjacent normal tissue samples, did not observe a significant change (delta beta = 0.0121, *p* = 0.6772). The small sample size limits the precision of this assessment; this result is inconclusive for direct-site validation rather than evidence that no difference exists. In GSE30601, regional methylation assessment in the HLA-DMA/HLA-DMB/BRD2 region based on platform gene annotation revealed that 3 out of 6 regionally covered CpGs reached FDR < 0.05. Since the GSE30601 platform did not cover cg24290947, these results do not provide direct evidence for the CpG-level effect of cg24290947; these regional observations cannot substitute for direct replication of the index CpG. The complete results of external methylation exploration are presented in Supplementary Table S8. Collectively, the evidence for cg24290947 as a direct CpG-level tumor-normal difference did not reach statistical significance, whereas methylation signals in this region were observed at both the exposure and regional levels.

### 3.5. External Expression Datasets Supported an MHC-II Antigen-Presentation Background in Gastric Cancer

**Figure 6:**
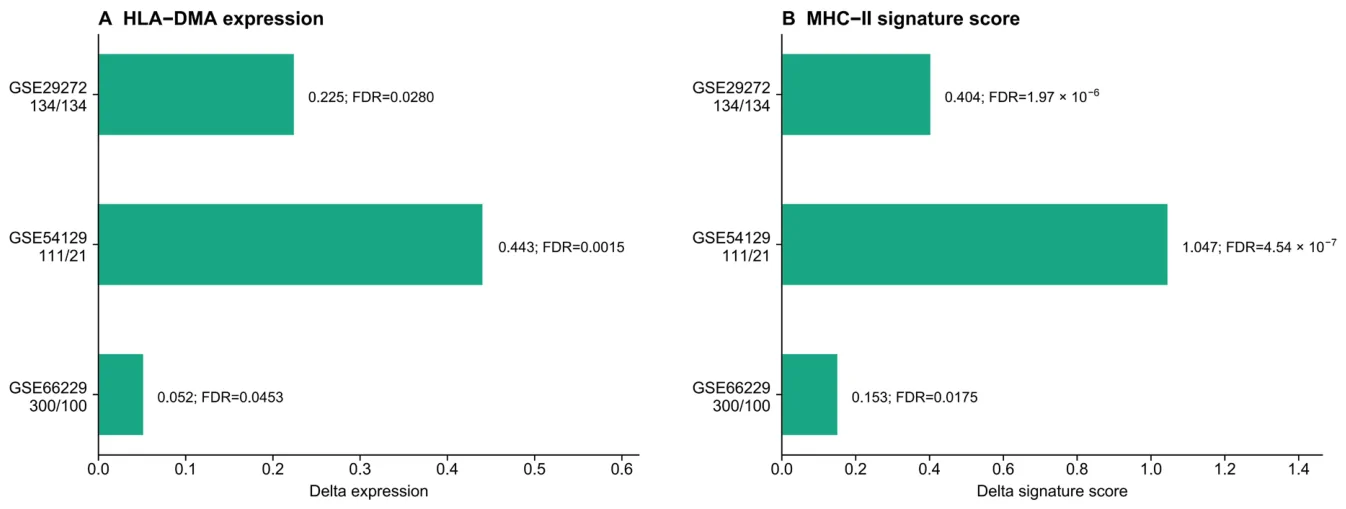
HLA-DMA expression and MHC-II signature scores in external gastric cancer expression cohorts. (**A**) Mean difference (delta) in HLA-DMA expression between tumor and non-tumor groups across three GEO datasets. (**B**) Mean difference (delta) in the MHC-II mean z-score signature between tumor and non-tumor groups. Delta represents the between-group mean difference in expression values or MHC-II signature scores, and is not directly equivalent to log_2_ fold-change unless the source matrix is already on a log_2_ scale.

To examine the transcriptional activity of HLA-DMA, the gene annotated to cg24290947, and its associated MHC-II antigen-presentation pathway in gastric cancer tissues, three external GEO expression cohorts were further utilized for expression context validation. The results showed that HLA-DMA expression tended to be higher in tumor than in normal tissues across multiple external expression cohorts (GSE29272: delta = 0.225, FDR = 0.0280; GSE54129: delta = 0.443, FDR = 0.0015; GSE66229: delta = 0.052, FDR = 0.0453). The MHC-II mean z-score signature also showed a tumor-higher-than-normal trend across the three cohorts (GSE29272: delta = 0.404, FDR = 1.97 × 10^−6^; GSE54129: delta = 1.047, FDR = 4.54 × 10^−7^; GSE66229: delta = 0.153, FDR = 0.0175) (Figure 6). The full results of external expression exploration are presented in Supplementary Table S9. Both HLA-DMA single-gene expression and the MHC-II pathway score showed consistent directions and reached statistical significance across the three external expression cohorts, collectively supporting a transcriptionally activated MHC-II antigen-presentation context in gastric cancer tissues. However, as these signals were derived from bulk tissues, they could not distinguish between the respective contributions of tumor cells and microenvironmental immune cells.

### 3.6. TCGA-STAD Combined Analysis Did Not Support Linear Methylation-Expression Coupling

To further evaluate the direct association of cg24290947 methylation with HLA-DMA expression and MHC-II pathway activity in matched tumor tissues, integrative analysis was performed using TCGA-STAD matched methylation-expression data, with a total of 354 primary tumor samples included. In the unadjusted analyses, cg24290947 was not significantly correlated with HLA-DMA expression (Spearman rho = −0.017, *p* = 0.7485) or the MHC-II signature (rho = 0.043, *p* = 0.4146). After stratification by the median cg24290947 methylation beta-value, no significant differences were observed between the high- and low-methylation groups in HLA-DMA expression (delta = −0.1079, *p* = 0.6509) or the MHC-II signature (delta = 0.0065, *p* = 0.7763), as detailed in Supplementary Table S10A.

In the composition-adjusted sensitivity analyses, the ImmuneScore-adjusted model showed a nominal inverse association between the methylation term and HLA-DMA expression (beta = −1.5743, SE = 0.4907, *p* = 0.0015, adjusted R^2^ = 0.4185), as well as a nominal inverse association with the MHC-II signature (beta = −0.6725, SE = 0.3067, *p* = 0.0290, adjusted R^2^ = 0.5551; the full results are provided in Supplementary Table S10B. However, these nominal associations were not consistently replicated across all compositional covariates: in the StromalScore-adjusted model, the methylation term was not significant for either HLA-DMA (*p* = 0.156) or the MHC-II signature (*p* = 0.555); and in the TumorPurity-adjusted model, the HLA-DMA association (*p* = 0.0139) was no longer significant after excluding high-influence samples above the Cook’s distance threshold (*p* = 0.0882), while the corresponding association for the MHC-II signature was also not significant (*p* = 0.0960), as shown in Supplementary Table S10B. Thus, among the six composition-adjusted models, three methylation terms reached nominal significance (*p* < 0.05), but the results depended on the adjustment model; the purity-adjusted HLA-DMA association no longer met nominal significance after high-influence observations were excluded.

Therefore, the unadjusted and composition-adjusted analyses did not reveal a stable linear association between cg24290947 methylation and HLA-DMA expression or the MHC-II signature in matched gastric cancer tissues. Although the nominal signals observed after ImmuneScore adjustment suggested that immune cell composition might modify this association, this effect was not consistently replicated across different compositional covariates. Overall, these observations do not support a simple linear regulatory model and do not identify the cell type responsible for the bulk-tissue associations.

### 3.7. Integrated Evidence Classification Supports cg24290947/HLA-DMA as a Regional Methylation Anchor

**Figure 7:**
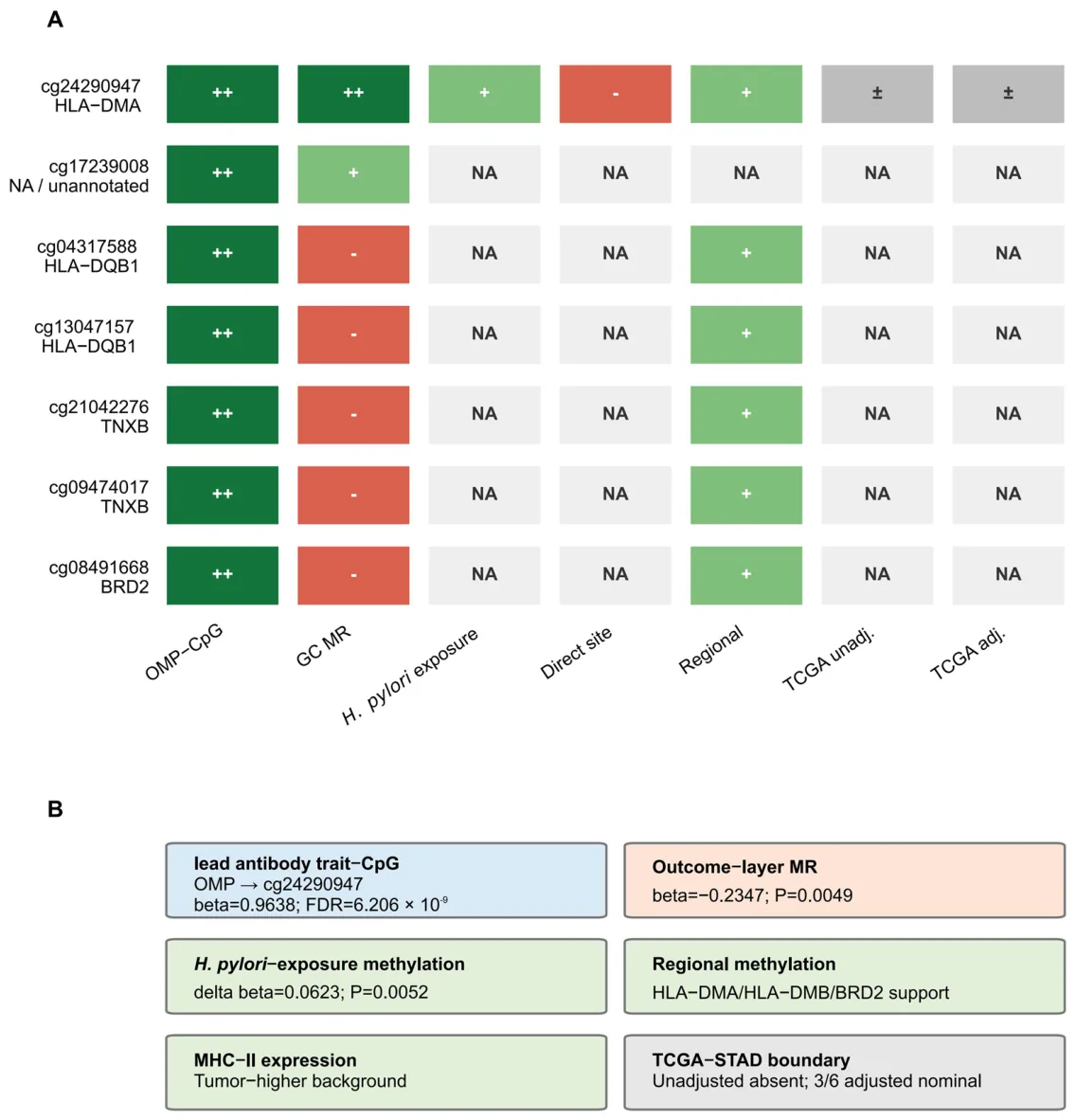
Integrated evidence matrix for the seven candidate CpGs. (**A**) Qualitative grading of the seven candidate CpGs and their annotated genes across five evidence layers: lead antibody trait discovery, CpG-to-gastric cancer MR, external methylation, external expression, and TCGA-STAD sensitivity. Evidence levels are denoted as “++” (FDR-supported primary evidence), “+” (nominal/regional/directional support), “±” (mixed/borderline), “–” (evaluated but not supportive), and “NA” (not evaluated/unavailable). (**B**) Summary of the evidence chain for cg24290947/HLA-DMA, illustrating the main quantitative evidence from OMP antibody response to gastric cancer risk: OMP antibody trait–CpG association; outcome-layer MR support; *H. pylori* exposure-related methylation support; regional methylation signatures (HLA-DMA/HLA-DMB/BRD2); and MHC-II expression background. TCGA-STAD methylation–expression coupling provided only borderline evidence and is not included among the positive evidence layers.

After completing lead antibody trait discovery, CpG-to-gastric cancer MR, external methylation evaluation, external expression evaluation, and TCGA-STAD unadjusted/composition-adjusted sensitivity analyses, we performed integrated evidence classification for the candidate CpG sites. The seven CpGs were summarized descriptively across distinct evidence layers rather than combined into a pooled effect or common testing family, with the complete evidence matrix provided in Supplementary Table S11 (Figure 7A). Among these, cg24290947/HLA-DMA had already been identified as an outcome-layer supported candidate in the outcome-layer MR. On this basis, we further integrated multiple layers of evidence—including *H. pylori* antibody-related methylation, gastric cancer tissue methylation, MHC-II expression background, and TCGA-STAD methylation–expression coupling—to assess whether this candidate site could be interpreted as a regional methylation anchor.

As shown in Figure 7A, the seven OMP-related candidate CpG sites (cg24290947, cg17239008, cg04317588, cg13047157, cg21042276, cg09474017, and cg08491668) and their annotated genes (HLA-DMA, HLA-DQB1, TNXB, and BRD2) were evaluated using a five-tier evidence classification system (“++”, “+”, “±”, “–”, and “NA”), with cg24290947/HLA-DMA receiving consistent support across multiple exploration layers. Figure 7B further focused on the complete evidence chain from OMP antibody response to gastric cancer risk for cg24290947/HLA-DMA, summarizing the following key findings: (1) the association between OMP antibody trait and cg24290947 methylation (beta = 0.9638; adjusted *p* = 6.206 × 10^−9^ across 652 evaluated OMP-CpG associations); (2) outcome-layer support from CpG-to-gastric cancer MR (beta = −0.2347, raw *p* = 0.0049); (3) external *H. pylori* exposure-related methylation support (delta beta = 0.0623, *p* = 0.0052); (4) regional methylation support involving HLA-DMA, HLA-DMB, and BRD2; and (5) elevated HLA-DMA/MHC-II-related expression background in external gastric cancer expression cohorts; Of note, (6)TCGA-STAD paired methylation–expression analysis showed that neither unadjusted correlation analysis nor high- versus low-methylation group comparisons supported a stable linear coupling between cg24290947 methylation and HLA-DMA expression or MHC-II signature. Among the six composition-adjusted models (with HLA-DMA expression and MHC-II signature as two endpoints, each adjusted for ImmuneScore, StromalScore, or ESTIMATE-derived inferred tumor purity), three models achieved nominal significance for the methylation term (*p* < 0.05); however, this result was not consistently replicated across different covariate models, and the TumorPurity-adjusted HLA-DMA association no longer reached nominal significance after excluding high-impact samples (*p* = 0.0882). Therefore, this coupling evidence is best interpreted as model-dependent borderline support. The qualitative grades reflect module-specific evidence and do not imply a direct regulatory or biomarker role. Overall, cg24290947/HLA-DMA is better positioned as a regional methylation anchor within the MHC/HLA-DMA locus than as a single-site linear expression regulator.

### 3.8. Targeted Functional Annotation Supported an Antigen-Presentation Immune Context

**Figure 8:**
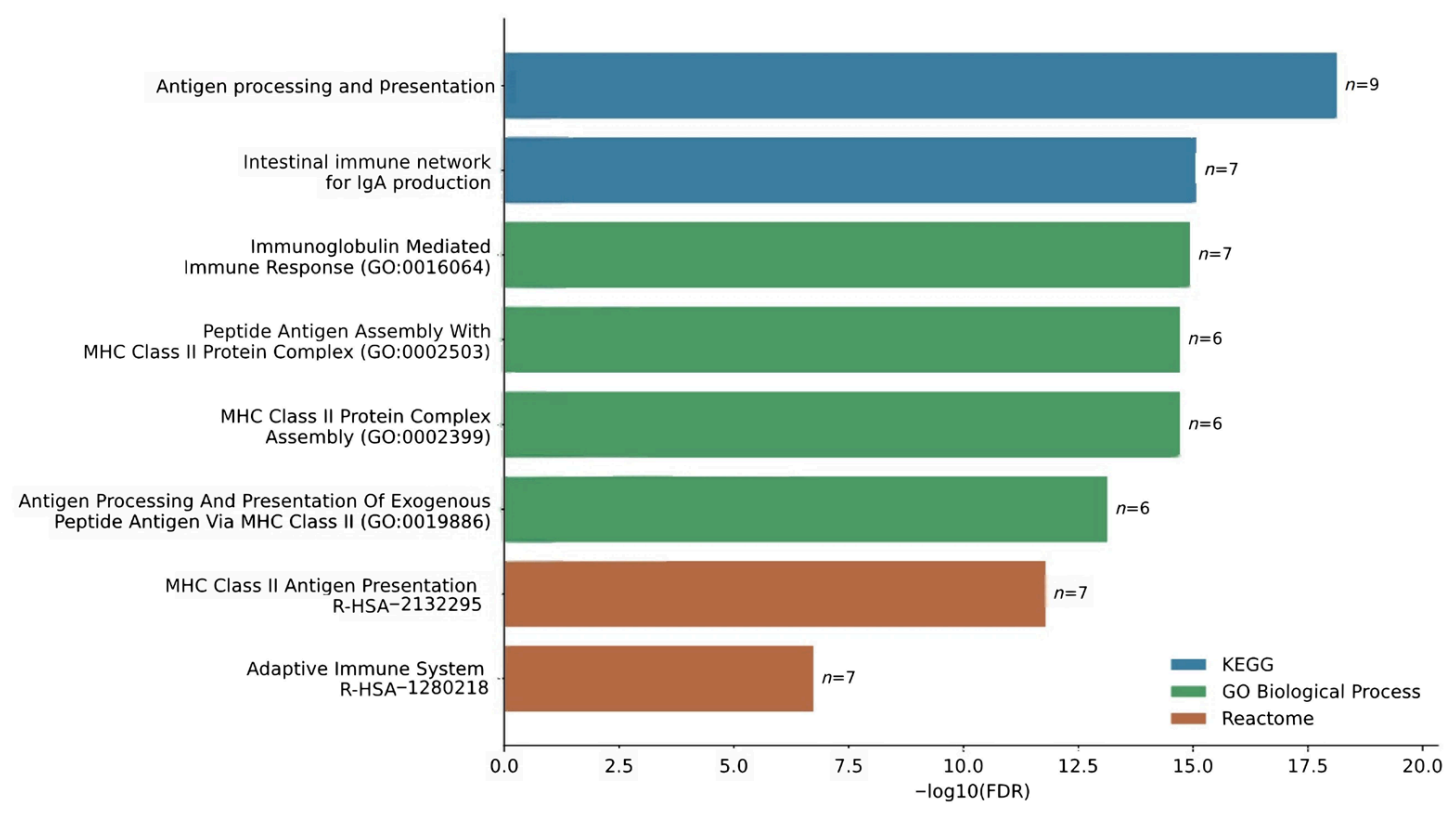
Targeted functional annotation of MHC/HLA region candidate genes. The horizontal axis represents -log_10_(FDR), with the number of input genes annotated to the pathway labeled at the end of each bar. Colors distinguish pathway databases (blue: KEGG; green: GO Biological Process; brown: Reactome). Representative terms related to antigen processing and presentation, MHC-II peptide loading, adaptive immunity, and mucosal immunity are displayed.

To assess whether the cg24290947/HLA-DMA regional methylation anchor involves antigen presentation-related pathways, targeted functional annotation was performed on genes in the candidate region, with the complete enrichment results provided in Supplementary Table S12. Using HLA-DMA/MHC-II-related genes and MHC region candidate genes as input, GO/KEGG/Reactome enrichment analysis revealed that this input gene set was significantly enriched for terms related to antigen processing and presentation, MHC class II peptide loading, adaptive immune response, and mucosal immune context. Representative terms included KEGG antigen processing and presentation (FDR = 7.05 × 10^−19^), KEGG intestinal immune network for IgA production (FDR = 8.13 × 10^−16^), Reactome MHC Class II Antigen Presentation (FDR = 1.56 × 10^−12^), and GO antigen processing and presentation of exogenous peptide antigen via MHC class II (FDR = 7.03 × 10^−14^) (Figure 8). Given that the input gene set already contained MHC-II and regional candidate genes, these enrichment results provide functional context for the candidate region rather than serving as independent confirmation that cg24290947 directly alters HLA-DM activity or antigen-presentation function. Collectively, these enrichment results provided pathway-level support for the immune-related functional orientation of the cg24290947/HLA-DMA regional methylation anchor.

## 4. Discussion

Host antibody response to *H. pylori* is an important manifestation of inter-individual differences in post-infection immune responses. *H. pylori* infection is a major infectious risk factor for gastric cancer [25], but its carcinogenic effects depend not only on infection status but also on host immune response patterns and epigenetic alterations [1]. *H. pylori* can induce DNA methylation changes in the gastric mucosa [26], generating an epigenetic field effect associated with gastric cancer risk [27], and these methylation changes have been linked to molecular subtyping, early biomarkers, and clinicopathological features [26]. Several studies have confirmed that *H. pylori* infection-induced host methylation alterations are associated with gastric cancer [28,29]. However, integrated evidence remains insufficient to elucidate how host antibody responses to distinct *H. pylori* antigens differentially influence methylation changes and are associated with gastric cancer risk.

In this study, MR analysis of host antibody responses to *H. pylori* antigens first identified OMP as the lead antibody target associated with gastric cancer risk (OR = 1.186, *p* = 4.08 × 10^−4^). Subsequently, through lead antibody-associated CpG discovery and outcome-layer MR analysis, cg24290947, annotated to the HLA-DMA gene in the MHC region, was identified as a methylation site with a potential association with gastric cancer risk (beta = −0.2347, FDR = 0.0097). External methylation exploration showed that cg24290947 methylation was significantly elevated in *H. pylori*-positive gastric mucosa (delta beta = 0.0623, *p* = 0.0052). In the small paired cohort (GSE164988), the direct CpG-level tumor-normal methylation difference at this site did not reach significance (delta beta = 0.0121, *p* = 0.6772). In addition, methylation perturbations in the HLA-DMA/MHC neighboring region were observed in gastric cancer (3/6 CpGs, FDR < 0.05). Notably, both HLA-DMA and the MHC-II signature showed a tumor-higher direction across three independent gastric cancer cohorts (all FDR < 0.05). However, neither the unadjusted nor the composition-adjusted TCGA-STAD analyses supported a simple linear regulatory relationship between cg24290947 methylation and HLA-DMA expression or the MHC-II signature, and the directions of the OMP antibody trait→gastric cancer (positive), OMP antibody trait→cg24290947 methylation (positive), and cg24290947→gastric cancer (inverse) associations did not support a conventional positive mediating pathway [30]. This directional pattern may reflect the influence of linkage disequilibrium (LD) in this region on the OMP genetic instruments, causing them to tag nearby methylation signals [31,32]. Therefore, cg24290947 is better interpreted as a regional methylation signal rather than a direct mediator. Collectively, the multi-layer evidence suggests that cg24290947/HLA-DMA may function as a regional epigenetic anchor within the MHC/HLA-DMA region involved in gastric cancer risk regulation. Such context-dependent regulation is consistent with previous observations in fibroblasts, where the regulatory relationship between DNA methylation and expression is inherently context-dependent and nonlinear, governed by chromatin state rather than genomic location [33]. This process may involve multiple complex mechanisms, including regional chromatin remodeling, enhancer-promoter interactions, or alternative splicing [34,35].

The MHC region is the most densely immune-related gene cluster in the human genome, and the MHC molecules it encodes serve as core components for the immune system to recognize “self” versus “non-self” antigens. Based on structural differences, MHC molecules are divided into class I and class II, with MHC class II molecules primarily responsible for presenting antigenic peptides derived from extracellular pathogens (e.g., *H. pylori*) to CD4^+^ T cells, thereby initiating adaptive immune responses [36]. The HLA-DMA gene is located within the MHC class II region and encodes the α subunit of the HLA-DM complex. Unlike other MHC molecules, HLA-DM does not directly bind antigenic peptides but functions as a “peptide loading catalyst”: newly synthesized MHC class II molecules have their peptide-binding grooves occupied by an inert fragment, the class II-associated invariant chain peptide (CLIP), before antigen loading. The core function of HLA-DM is to remove CLIP and facilitate the efficient loading of high-affinity antigenic peptides onto MHC class II molecules [37,38]. HLA-DMA is an indispensable functional gene in the MHC class II peptide loading process [39]. As a DNA methylation site, cg24290947 is genomically annotated to the HLA-DMA locus, suggesting that this region may participate in the MHC class II antigen presentation pathway. However, the lack of a stable linear relationship between methylation at this site and HLA-DMA expression indicates that its functional relevance—if any—is unlikely to be mediated through simple cis-transcriptional regulation of the annotated gene. Instead, methylation perturbations in the vicinity of the MHC/HLA-DMA locus, along with the overall elevated expression of MHC class II pathway genes in tumor tissues, suggest that cg24290947 methylation may reflect broader epigenomic remodeling at this site in response to *H. pylori*-related immune pressure. Overall, the lack of a stable linear association between cg24290947 methylation and HLA-DMA expression does not support a cis-transcriptional regulatory role for this site. Its functional relevance awaits further validation through cell-type-resolved methylation profiling, targeted epigenome editing, or single-cell multi-omics analyses.

Methodologically, this study integrated MR analysis with external stratified evaluation, allowing conclusions to be cross-validated across both genetic instrument-based inference and external generalizability. Several limitations of this study should be acknowledged. First, the MR and mQTL analyses relied on publicly available summary-level data, precluding access to individual-level infection status or treatment information. Second, the mQTL instruments were derived from peripheral blood rather than gastric mucosa; although external methylation data from gastric tissues (GSE99553) partially addressed this gap, blood-derived mQTLs may capture systemic rather than tissue-specific effects, and the extent of blood–gastric methylation concordance at the MHC locus remains incompletely characterized. Third, the findings are based primarily on European populations and may not be generalizable to East Asian populations, given substantial differences in *H. pylori* virulence and gastric cancer epidemiology between these populations. Fourth, the external GEO datasets were derived from different platforms and could not be merged; moreover, methylation and expression data lacked direct pairing, limiting inferences about regulatory relationships, while the small sample size of GSE164988 precludes definitive conclusions and GSE30601 did not cover the index CpG. Fifth, formal colocalization analysis in the HLA/MHC region was not performed due to complex LD and the lack of confirmed complete regional summary statistics; therefore, linked but distinct signals cannot be excluded. Sixth, given that the antibody traits were identified using pre-specified instrument selection threshold and the candidate CpGs underwent multi-stage screening, the reliability of instrument strength and the validity of multiplicity correction may be affected. Despite these limitations, cg24290947, as an MHC region methylation anchor associated with *H. pylori* OMP antibody response and gastric cancer risk, remains a hypothesis-generating candidate. The present study does not provide evidence for predictive performance, clinical risk stratification, or therapeutic benefit, and these findings await further validation.

## 5. Conclusions

This study identified an OMP antibody-associated methylation candidate at cg24290947/HLA-DMA and an inverse genetic association with gastric cancer risk. External tissue findings support regional context but do not establish a causal regulatory relationship with HLA-DMA. The genetic results are based on European-ancestry summary data and remain subject to limitations including instrument validity, tissue specificity, multiplicity, and regional LD. This candidate warrants independent biological and cross-ancestry investigation; its clinical predictive value has not been demonstrated.

## Data Availability

The original data presented in this study are openly available from publicly accessible repositories. The *H. pylori* antibody traits GWAS and gastric cancer GWAS summary statistics were sourced from the IEU OpenGWAS/MR-Base platform (available at: https://opengwas.io/ (accessed on 9 September 2026); API documentation: https://api.opengwas.io/api/ (accessed on 9 September 2026)). The mQTL summary statistics were obtained from the GoDMC database (available at: https://mqtldb.godmc.org.uk/ (accessed on 9 September 2026); https://api.opengwas.io/api/ (accessed on 9 September 2026)). External methylation and expression validation data were sourced from the GEO, with accession numbers GSE99553, GSE164988, GSE30601, GSE29272, GSE54129, and GSE66229. TCGA-STAD methylation and expression data were accessed via the UCSC Xena platform (https://xena.ucsc.edu/ (accessed on 9 September 2026); https://api.opengwas.io/api/ (accessed on 9 September 2026); GDC Xena hub: https://gdc.xenahubs.net/ (accessed on 9 September 2026)). The expression matrix used was TCGA-STAD.star_counts.tsv and the methylation matrix was TCGA-STAD.methylation450.tsv. Functional annotation analysis was performed using the Enrichr online tool (https://maayanlab.cloud/Enrichr/ (accessed on 9 September 2026)). All datasets analyzed during this study are included in this published article and its Supplementary Information files.

## Supplementary Materials

The following supporting information can be downloaded at: https://www.mdpi.com/article/10.3390/genes17091125/s1, Table S1: Data sources and analytical roles; Table S2: Antibody-trait instrument counts and F-statistic information; Table S3A: OMP antibody trait-CpG screening summary and exploratory tier rules; Table S3B: All 652 evaluated OMP antibody trait-CpG associations; Table S4: Genes used for targeted functional annotation; Table S5: *H. pylori* antibody-trait-to-gastric-cancer MR results; Table S6: Seven CpGs assembled for integrated presentation; Table S7A: Seven displayed CpG-to-gastric-cancer MR estimates and index-CpG instrument QC; Table S7B: All 15 unique CpGs evaluated for gastric cancer; Table S8: External methylation results; Table S9: External expression results; Table S10A: TCGA-STAD unadjusted contextual analyses; Table S10B: TCGA-STAD composition-adjusted sensitivity analyses; Table S11: Integrated evidence matrix for the seven displayed CpGs; Table S12: Targeted functional annotation results. Supplementary Methods: Detailed screening rules for lead antibody trait-associated CpG prioritization; the STROBE-MR checklist is provided as a separate file.
