# Supplementary material for: H. pylori Outer Membrane Protein Antibody Response, MHC/HLA-DMA Methylation, and Gastric Cancer Risk: A Multi-Layer Evidence Integration Study Using Mendelian Randomization
Source: Genes (Basel). 2026 Sep 15;17(9):1125. doi: 10.3390/genes17091125 (PMC13606778; doi:10.3390/genes17091125)
Supplement: Supplementary file 1 [file genes-17-01125-s001.zip › STROBE-MR_checklist.pdf]

STROBE-MR checklist of recommended items to address in reports of Mendelian randomization studies

Item No.	Section	Checklist item	Page No.	Relevant text from manuscript
1	**TITLE and ABSTRACT**	Indicate Mendelian randomization (MR) as the study's design in the title and/or the abstract if that is a main purpose of the study	Title / Abstract	**tile:** "*H. pylori* outer membrane protein antibody response, MHC/HLA-DMA methylation, and Gastric Cancer Risk: a multi-layer evidence integration study using Mendelian randomization."<br>**Abstract:** "Using a forward two-sample Mendelian randomization (MR) design, we integrated summary-level data from mQTLs, external cohorts, TCGA-STAD, and functional annotations through a three-stage analytical framework..."
	**INTRODUCTION**			
2	**Background**	Explain the scientific background and rationale for the reported study. What is the exposure? Is a potential causal relationship between exposure and outcome plausible? Justify why MR is a helpful method to address the study question	Introduction	"Although observational studies have revealed close associations among H. pylori infection, DNA methylation, and gastric cancer, the existing evidence is largely limited to descriptive correlational analyses and lacks a genetically informed framework." "To address these limitations, this study employs a forward two-sample MR approach, using genetic variants associated with host antibody responses to H. pylori antigens as exposure instruments and mQTL as mediator instruments."

3	**Objectives**	State specific objectives clearly, including pre-specified causal hypotheses (if any). State that MR is a method that, under specific assumptions, intends to estimate causal effects	Introduction	"The specific aims include: (1) identifying the H. pylori antigen whose antibody response is most significantly associated with gastric cancer risk; (2) identifying CpG sites associated with this antibody response and evaluating their genetic associations with gastric cancer through multi-stage outcome assessment; and (3) exploring tissue-level support for methylation signals in the candidate regions using external independent data."
	**METHODS**			
4	**Study design and data sources**	Present key elements of the study design early in the article. Consider including a table listing sources of data for all phases of the study. For each data source contributing to the analysis, describe the following:	Methods 2.1–2.2	"The study combined publicly available summary-level genetic data, mQTL, and external multi-layer data to construct a hierarchical evidence framework, following the logic of forward two-sample MR and a two-step MR design. The analytical workflow comprised four stages." "Summary-level GWAS data for H. pylori antigen antibody traits were obtained from the IEU OpenGWAS/MR-Base platform, all derived from European populations."
	a)	Setting: Describe the study design and the underlying population, if possible. Describe the setting, locations, and relevant dates, including periods of recruitment, exposure, follow-up, and data collection, when available.	Methods 2.2	The GWAS summary statistics used in this study were obtained from publicly available datasets: the H. pylori antibody trait GWAS (IEU OpenGWAS/MR-Base platform) and the gastric cancer outcome GWAS (ebi-a-

			GCST90018849), both derived from European-ancestry populations. mQTL data were obtained from the GoDMC consortium, a large-scale blood-based methylation QTL resource integrating 32,851 participants of European ancestry. The original GWAS and QTL studies were conducted in diverse settings, and detailed information regarding recruitment, exposure assessment, and data collection is available in the original publications (Butler-Laporte et al., 2020 [15]; Sun et al., 2023; Min et al., 2021 [16]). All data used in this study are summary-level statistics; no individual-level participant data were accessed, and therefore no additional recruitment or follow-up periods apply to the present analysis.
b)	Participants: Give the eligibility criteria, and the sources and methods of selection of participants. Report the sample size, and whether any power or sample size calculations were carried out prior to the main analysis	Methods 2.2, 2.5–2.6	"The six included H. pylori antigens and their corresponding OpenGWAS IDs were as follows: CagA (n=985), Catalase (n=1,558), GroEL (n=2,716), OMP (n=2,640), UREA (n=2,251), and VacA (n=1,571). Gastric cancer outcome data… comprising 1,029 cases and 475,087 controls (total n=476,116)." "GoDMC is a large-scale resource based on peripheral-blood DNA methylation, integrating data from 32,851 participants." "GSE29272 contained 134 pairs of tumor-normal expression samples. GSE54129 contained 111

			gastric cancer tissues and 21 non-cancerous gastric tissues. GSE66229 contained 300 tumor and 100 normal samples." No power calculations were performed prior to the main analysis.
c)	Describe measurement, quality control and selection of genetic variants	Methods 2.3	"Instrumental variables were extracted from the exposure GWAS at a threshold of $P<5\times10^{-6}$ and LD-clumped using an r^2 threshold of 0.001 within a 10,000-kb window with a European reference panel." "Instrument strength was assessed using the F-statistic, with $F>10$ considered indicative of low risk of weak instrument bias."
d)	For each exposure, outcome, and other relevant variables, describe methods of assessment and diagnostic criteria for diseases	Methods 2.2，2.5–2.6	"In the originating antibody GWAS, quantitative antibody Mean Fluorescence Intensity (MFI) analyses were restricted to participants exceeding the relevant seropositivity threshold; the quantitative phenotypes were standardized to mean 0 and standard deviation 1 and analyzed using a fastGWA mixed model." External methylation and expression datasets were analyzed using processed matrices from GEO.
e)	Provide details of ethics committee approval and participant informed consent, if relevant	Not applicable	All data used in this study are publicly availa [illegible] published GWAS and QTL studies; no individ [illegible]

5	**Assumptions**		Explicitly state the three core IV assumptions for the main analysis (relevance, independence and exclusion restriction) as well assumptions for any additional or sensitivity analysis	Methods 2.3	This MR framework relies on the three core instrumental variable assumptions: (1) the genetic variants are associated with the exposure (relevance); (2) they are not associated with confounders of the exposure–outcome relationship (independence); and (3) they affect the outcome only through the exposure (exclusion restriction).
6	**Statistical methods: main analysis**		Describe statistical methods and statistics used		
		a)	Describe how quantitative variables were handled in the analyses (i.e., scale, units, model)	Methods 2.3	"In the originating antibody GWAS, quantitative antibody MFI analyses were restricted to participants exceeding the relevant seropositivity threshold; the quantitative phenotypes were standardized to mean 0 and standard deviation 1..."
		b)	Describe how genetic variants were handled in the analyses and, if applicable, how their weights were selected	Methods 2.3	"Instrumental variables were extracted from the exposure GWAS at a threshold of $P<5\times10^{-6}$ and LD-clumped using an r^2 threshold of 0.001 within a 10,000-kb window with a European reference panel."
		c)	Describe the MR estimator (e.g. two-stage least squares, Wald ratio) and related statistics. Detail the included covariates and, in case of two-sample MR, whether the same covariate set was used for adjustment in the two samples	Methods 2.3	"The inverse variance weighted (IVW) method was used as the primary estimator." "MR-Egger, weighted median, simple mode, and weighted mode estimates were used as sensitivity analyses when the available instrument

				number permitted." "For multi-instrument analyses, IVW was used as the primary estimator; for single-instrument analyses, the Wald ratio was applied." Two-sample MR used summary-level data; covariates were adjusted within each original GWAS.
		d) Explain how missing data were addressed	Not applicable	No missing data were imputed; analyses were performed on available harmonized summary statistics.
		e) If applicable, indicate how multiple testing was addressed	Methods 2.3, 2.9	"Multiple comparisons were corrected using the Benjamini-Hochberg FDR method, with FDR < 0.05 considered the threshold for statistical significance." "Adjustment was applied within the corresponding analysis families rather than across different analysis families."
7	**Assessment of assumptions**	Describe any methods or prior knowledge used to assess the assumptions or justify their validity	Methods 2.3	"Heterogeneity was assessed using Cochran's Q test, directional horizontal pleiotropy using the MR-Egger intercept test, and MR-PRESSO global testing when applicable. Instrument strength for traceable instrument sets was assessed using $F = (beta\_exposure / SE\_exposure)^2$."
8	**Sensitivity analyses and additional analyses**	Describe any sensitivity analyses or additional analyses performed (e.g. comparison of effect estimates from different approaches, independent replication, bias analytic techniques, validation of instruments, simulations)	Methods 2.3, 2.5–2.8	"MR-Egger, weighted median, simple mode, and weighted mode estimates were used as

				sensitivity analyses." External methylation and expression datasets were used for independent contextual evaluation. TCGA-STAD composition-adjusted sensitivity analyses were performed. Targeted functional annotation was conducted via Enrichr.
9	**Software and pre-registration**			
		a) Name statistical software and package(s), including version and settings used	Methods 2.9	"MR analyses were performed using R 4.5.0, TwoSampleMR 0.7.0, and the MR-Base workflow." "Table preparation, external cohort statistical analyses, and the TCGA-STAD methylation combined analysis were performed using Python 3.12.10 with the pandas, numpy, scipy, statsmodels, and matplotlib packages."
		b) State whether the study protocol and details were pre-registered (as well as when and where)	Not applicable	This study was not pre-registered.
	**RESULTS**			
10	**Descriptive data**			
		a) Report the numbers of individuals at each stage of included studies and reasons for exclusion. Consider use of a flow diagram	Results 3.1–3.2	"Among the six H. pylori antibody traits, OMP showed the most robust positive association... UREA did not yield a valid IVW estimate." "A total of 652 CpGs were detected... of which 519 reached FDR < 0.05... exploratory tier-based rules retained 132 candidates (109 Tier2

				and 23 TierS)... seven CpGs were assembled for integrated presentation."
	b)	Report summary statistics for phenotypic exposure(s), outcome(s), and other relevant variables (e.g. means, SDs, proportions)	Results 3.1–3.5	Results sections report OR, beta, 95% CI, P values, FDR, delta beta, F statistics for all key variables.
	c)	If the data sources include meta-analyses of previous studies, provide the assessments of heterogeneity across these studies	Not applicable	This study did not include meta-analyses of previous studies.
	d)	For two-sample MR:<br>i. Provide justification of the similarity of the genetic variant-exposure associations between the exposure and outcome samples<br>ii. Provide information on the number of individuals who overlap between the exposure and outcome studies	Methods 2.2	i. "Summary-level GWAS data for H. pylori antigen antibody traits were obtained from the IEU OpenGWAS/MR-Base platform, all derived from European populations." "Gastric cancer outcome data were obtained from ebi-a-GCST90018849 (European population)." ii. The exposure and outcome GWAS are from independent non-overlapping samples (two-sample MR design).
11	**Main results**			
	a)	Report the associations between genetic variant and exposure, and between genetic variant and outcome, preferably on an interpretable scale	Results 3.1, 3.3	"OMP showed the most robust positive association with gastric cancer risk (IVW OR = 1.186, 95% CI: 1.079–1.304, raw P = 4.08e-04)." "cg24290947/HLA-DMA showed the strongest genetic association (beta = −0.2347, 95% CI: −0.3980 to −0.0713, raw P = 0.0049)."
	b)	Report MR estimates of the relationship between exposure and outcome, and the measures of uncertainty from the MR analysis, on an interpretable scale, such as odds ratio or relative risk per SD difference	Results 3.1, 3.3	"OR = 1.186, 95% CI: 1.079–1.304, raw P = 4.08e-04." "beta = −0.2347, 95% CI: −0.3980 to −0.0713, raw P = 0.0049."
	c)	If relevant, consider translating estimates of relative risk into absolute risk for a meaningful time period	Not applicable	Not applicable for this MR study.

		d)	Consider plots to visualize results (e.g. forest plot, scatterplot of associations between genetic variants and outcome versus between genetic variants and exposure)	Results	Figures 2, 3, 4, 5, 6, 7, 8 provide forest plots ar evidence integration.
12	**Assessment of assumptions**				
		a)	Report the assessment of the validity of the assumptions	Results 3.1, 3.3	"Sensitivity analyses supported the robustness... with no significant heterogeneity detected (Cochran's Q P = 0.2159) or directional horizontal pleiotropy (MR-Egger intercept P = 0.2247; MR-PRESSO global test P = 0.208)." "Cochran's Q P = 0.5054; MR-Egger intercept P = 0.7320."
		b)	Report any additional statistics (e.g., assessments of heterogeneity across genetic variants, such as I^2, Q statistic or E-value)	Results 3.1, 3.3	"Cochran's Q P = 0.2159; MR-Egger intercept P = 0.2247; MR-PRESSO global test P = 0.208." "Cochran's Q P = 0.5054; MR-Egger intercept P = 0.7320." F statistics reported for instrument strength.
13	**Sensitivity analyses and additional analyses**				
		a)	Report any sensitivity analyses to assess the robustness of the main results to violations of the assumptions	Results 3.1, 3.3	"MR-Egger, weighted median, simple mode, and weighted mode estimates were used as sensitivity analyses." Sensitivity analyses results reported in text and supplementary tables.

		b)	Report results from other sensitivity analyses or additional analyses	Results 3.4–3.8	External methylation (3.4), external expression (3.5), TCGA-STAD (3.6), integrated evidence classification (3.7), and targeted functional annotation (3.8) results are reported.
		c)	Report any assessment of direction of causal relationship (e.g., bidirectional MR)	Not applicable	Bidirectional MR was not performed in this study.
		d)	When relevant, report and compare with estimates from non-MR analyses	Not applicable	No non-MR analyses were performed.
		e)	Consider additional plots to visualize results (e.g., leave-one-out analyses)	Not applicabl	Leave-one-out analyses were not performed
	**DISCUSSION**				
14	**Key results**		Summarize key results with reference to study objectives	Discussion	In this study, MR analysis of host antibody responses to H. pylori antigens first identified OMP as the lead antibody target associated with gastric cancer risk... cg24290947/HLA-DMA may function as a regional epigenetic anchor..."
15	**Limitations**		Discuss limitations of the study, taking into account the validity of the IV assumptions, other sources of potential bias, and imprecision. Discuss both direction and magnitude of any potential bias and any efforts to address them	Discussion	Six limitations are discussed: (1) summary-level data limitations; (2) blood-derived mQTLs vs. gastric mucosa; (3) European ancestry; (4) GEO platform heterogeneity; (5) no colocalization analysis; (6) pre-specified instrument selection and multiplicity.
16	**Interpretation**				
		a)	Meaning: Give a cautious overall interpretation of results in the context of their limitations and in comparison with other studies	Discussion	"cg24290947 is better interpreted as a regional methylation signal rather than a direct mediator." "The present study does not provide evidence for

				predictive performance, clinical risk stratification, or therapeutic benefit."
		b) Mechanism: Discuss underlying biological mechanisms that could drive a potential causal relationship between the investigated exposure and the outcome, and whether the gene-environment equivalence assumption is reasonable. Use causal language carefully, clarifying that IV estimates may provide causal effects only under certain assumptions	Discussion	As a DNA methylation site, cg24290947 is genomically annotated to the HLA-DMA locus, suggesting that this region may participate in the MHC class II antigen presentation pathway...... Its functional relevance awaits further validation through cell-type-resolved methylation profiling, targeted epigenome editing, or single-cell multi-omics analyses.
		c) Clinical relevance: Discuss whether the results have clinical or public policy relevance, and to what extent they inform effect sizes of possible interventions	Discussion / Conclusions	"cg24290947... remains a hypothesis-generating candidate. The present study does not provide evidence for predictive performance, clinical risk stratification, or therapeutic benefit." "Its clinical predictive value has not been demonstrated."
17	**Generalizability**	Discuss the generalizability of the study results (a) to other populations, (b) across other exposure periods/timings, and (c) across other levels of exposure	Discussion	"The findings are based primarily on European populations and may not be generalizable to East Asian populations, given substantial differences in H. pylori virulence and gastric cancer epidemiology between these populations."
	**OTHER INFORMATION**			
18	**Funding**	Describe sources of funding and the role of funders in the present study and, if applicable, sources of funding for the databases and original study or studies on which the present study is based	Declarations	Funding:National Natural Science Foundation of China (82274511, 82575002); Xiyuan Hospital Special Research Project (XYZX0204-03).

19	**Data and data sharing**	Provide the data used to perform all analyses or report where and how the data can be accessed, and reference these sources in the article. Provide the statistical code needed to reproduce the results in the article, or report whether the code is publicly accessible and if so, where	Declarations / Methods	Data access: All data from OpenGWAS, GoDMC, GEO, UCSC Xena, Enrichr. Code：Available from the corresponding author upon request.
20	**Conflicts of Interest**	All authors should declare all potential conflicts of interest	Declarations	Conflicts: The authors declare no conflicts of interest.
