# Supplementary material for: H. pylori Outer Membrane Protein Antibody Response, MHC/HLA-DMA Methylation, and Gastric Cancer Risk: A Multi-Layer Evidence Integration Study Using Mendelian Randomization
Source: Genes (Basel). 2026 Sep 15;17(9):1125. doi: 10.3390/genes17091125 (PMC13606778; doi:10.3390/genes17091125)
Supplement: Supplementary file 1 [file genes-17-01125-s001.zip › Supplementary Methods Detailed screening rules for lead antibody trait–associated CpG prioritization.pdf]

**Supplementary Methods: Detailed screening rules for lead antibody trait–associated CpG prioritization**

This supplementary method describes the detailed discovery-phase prioritization procedure for lead antibody trait–associated CpGs. The screening was exploratory and tier-based, using OMP-CpG association summary data, instrument quality, and genomic annotation information to progressively filter candidate CpG sites. Subsequent CpG-to-gastric cancer MR analyses and external validation results were not used as screening criteria in this phase.

**1. Screening overview**

Among the 652 evaluated OMP-CpG associations, Benjamini-Hochberg adjustment was applied across these 652 associations; 519 associations had FDR<0.05. Candidate retention was exploratory and tier-based rather than restricted to those 519 associations. The procedure retained 132 candidates, comprising 109 Tier2 and 23 TierS candidates, with no Tier1 candidate. The retained set was therefore not a strict subset of the FDR-supported associations. Missing sensitivity diagnostics were not interpreted as evidence that tests had been passed.

**2. Tier classification rules**

Classification was performed in the following order: TierS was evaluated first, followed by Tier1 and Tier2; records that did not meet these conditions were assigned to Tier3.

**TierS (special candidate):** Required one instrument, raw P<0.05, and available gene or positional annotation.

**Tier1 (high-confidence candidate):** Required FDR<0.05; at least three instruments; no single-instrument status; no recorded single-variant driver; and direction consistency ≥0.6 when that indicator was available. Under the recorded Tier1 quality-control rule, Cochran's Q, MR-Egger intercept and MR-PRESSO global P

values were each required to be at least 0.05 when available; missing diagnostic P values were permitted by the rule and were not treated as positive sensitivity evidence.

**Tier2 (moderate-confidence candidate):** Required raw $P<0.05$; at least two instruments; direction consistency ≥0.6 when available; and no recorded single-variant driver. FDR<0.05 and the additional Tier1 diagnostic conditions were not required.

**Direction consistency** was defined as the fraction of valid MR-method estimates sharing the sign of the first estimate and was unavailable when at most one valid method estimate existed.

**Leave-one-out analysis** required more than two instruments. When leave-one-out analysis was not performed, failed or produced no usable output, the driver flag could remain false and was not interpreted as evidence that the test had been passed. All 109 Tier2 candidates had two instruments, unavailable direction-consistency values and no eligibility for the recorded leave-one-out procedure.

**3. Shortlist formation and presentation set assembly**

Candidates classified as TierS, Tier1 or Tier2 were retained. Ranking and annotation information were subsequently used to construct an initial eight-CpG OMP shortlist; four VacA comparator candidates and a later three-CpG OMP evaluation were considered separately. Seven CpGs were subsequently assembled for integrated presentation. The final seven-CpG presentation set did not constitute a prospectively predefined seven-test MR family.
